# Study on the Relationship Between Uncertainty Tolerance and Positive Acceptance in Postoperative Patients With Cervical Cancer

**DOI:** 10.7759/cureus.54414

**Published:** 2024-02-18

**Authors:** Binbin Xu, Miaohong Qian, Lanfen Zhu, Zhaie Lu

**Affiliations:** 1 Gynecologic Oncology, Ningbo Women and Children's Hospital, Ningbo, CHN; 2 Gynecology, Ningbo Women and Children's Hospital, Ningbo, CHN; 3 Obstetrics and Gynaecology, Ningbo Women and Children's Hospital, Ningbo, CHN

**Keywords:** cervical cancer, postoperative, uncertainty tolerance, positive acceptance, correlation

## Abstract

Objective: The objective of this study is to clarify the correlation between uncertainty tolerance (UT) and positive acceptance (PA) in patients with cervical cancer (CC) after surgical treatment.

Methods: A total of 233 patients with CC who had undergone surgery were included and were scored on the UT Scale and Positive Acceptance Scale. In addition, patients were classified according to the length of stay ≥1 week and length of stay <1 week, and the UT score and satisfaction and enterprising score of the two groups were compared. This was performed in order to analyze the effect of length of hospital stay on UT and PA.

Results: The mean UT score of 233 patients was 3.74±0.34 and the mean PA score was 1.96±0.20, with a negative correlation and a significant correlation coefficient (r=-0.342, P 0.05). The UT score of post-operative CC patients with length of stay ≥1 week was significantly higher than that of patients with length of stay <1 week, P<0.05. The score of PA in patients with post-operative CC whose hospital stay was ≥1 week was significantly lower than for patients with hospital stays <1 week (P<0.05). UT was negatively correlated with PA in patients with hospital stays < 1 week (r=-0.358, P<0.05). There was a significant negative correlation between UT and PA in patients with hospital stay ≥1 week (r =-0.493, P<0.05). Increased hospitalization time correlated with increased scores of UT, with reductions in scores of PA.

Conclusion: Post-operative patients with CC had higher scores of UT and lower scores of PA, which were negatively correlated. Increased hospitalization time was linked to a detriment in patient UT and reduced PA. Targeted interventions to improve the level of UT and PA within postoperative CC cases should be developed.

## Introduction

Cervical cancer (CC) occurs at a rate of 15.36 cases/100,000 individuals in China reflecting a highly prevalent malignancy among Chinese females [1]. The prognosis for CC has risen significantly as a result of early screening becoming more accessible and advances in surgical procedures leading to quick diagnosis and treatment [2]. Notwithstanding, 29 - 38% of CC cases experience post-therapeutic relapses [3]. CC is mostly treated by radical surgery. The uterus is an important reproductive organ for women, and patients may lose their sense of identity after hysterectomy, which also leads to adverse psychological states in patients post-CC, such as anxiety, depression, and fear of cancer, and could also reduce individual uncertainty tolerance (UT).

UT primarily describes unique variations across patients regarding how they manage their prognosis, circumstances, mood swings, cognitive shifts, language, and behavior as CC cases. Numerous investigations demonstrated that people having decreased UT often perceive and appraise condition data and their surroundings adversely [4]. Such a pessimistic perspective might exacerbate physical/psychological stress, which would be harmful to the progression of the illness. UT may have a certain impact on post-operative patients with CC. Studies demonstrated that healthy people have higher UT levels than cancer patients [4].

A positive attitude is especially important after CC surgery. Patients could freely embrace their state of health and their surroundings and constructively react to stressful circumstances by adopting an accepting and entrepreneurial coping strategy [5]. Positive acceptance (PA) has been seen by Chinese academics as a crucial indicator of mental wellness. In addition, studies have shown that patients with CC scored worse than the healthy population regarding PA, with increased PA being linked to increased quality of life for CC patients. This also suggests that there may be a certain correlation between PA and UT post-CC surgery [6]. However, no reports have been reported so far.

This study analyzed possible associations across UT and PA scores of post-operative CC patients and further explored the correlation between UT and PA scores in post-operative CC patients with a differing length of hospital stay, in order to provide the basis for the prognosis and extended nursing of CC patients post-surgical treatment.

## Materials and methods

Experimental design

Between June 2018 and December 2020, 233 CC patients were selected during the inpatient period within a tertiary-care hospital in Ningbo.

Inclusion criteria were pathology-based CC diagnosis; CC stage ⅰ B - ⅱ A; major hysterectomy/pelvic and abdominal lymph node dissection; good literacy/understanding skills; full compliance and approval to participate in the investigation; no prior pre-operative chemo-/radio-therapy. Exclusion criteria consisted of respondents who did not complete the questionnaire; patients with other tumors; death or loss of follow-up.

Experimental protocol

Research Tools

Utilizing a questionnaire survey approach, this investigation probed age, marital status, form of medical insurance, cancer stage, and pathology-based classification, together with individual patient issues. This investigation additionally probed appropriate data contained within individual pertinent medical records. The "UT Scale" compiled by Zhihui Yang [7] is based on the characteristics of the Chinese population. Four parameters compose the scale: stress brought about by uncertainty, unjust future unpredictability, and events that have an adverse impact, as well as helplessness resulting from ambiguity. The Likert 5-level evaluation technique is used for an aggregate of 27 questions. Responses for every entry range from "1" (not at all like me) to "5" (exactly like me), with the overall score fluctuating between 27 and 135 points. The CC patient cohort decreases with increasing scale score. Every employed scale parameter has a test-retest reliability of 0.852～0.876.

This investigation employed the previously developed Positive Acceptance Scale [8] applied for Chinese cohorts. The employed scale incorporated 25 elements, divided into two categories: acceptance and positivity. It operated a Likert 5-level scoring approach, with an overall scoring ranging from 25 to 125 points. Individual element responses ranged from "1" (totally non-conforming) to "5" (entirely conforming). Both scale categories had a test-retest reliability of 0.831 and 0.853.

Analytical protocol

The authors conducted a questionnaire examination and scored the UT/Positive Acceptance Scales upon individual discharge day. Authors employed just one direction to describe the questionnaire completing process independently, once receiving informed consent. Authors reviewed medical records, filled with information from the survey participant, excluding certain details about the ailment. Authors reviewed and clarified the questionnaire for patients having difficulty filling it out independently, and subsequently filled it out on their own accord. A total of 256 questionnaires were collected, and the incomplete questionnaires were discarded. Altogether, this investigation obtained 233 valid questionnaires, with an effective rate of 91.02%.

Statistical analyses

IBM SPSS Statistics for Windows, Version 22 (Released 2013; IBM Corp., Armonk, New York, United States) was used for the statistical analysis. The measurement data were examined for normality, and those that passed muster were represented using the notation (mean ±standard deviation). The independent sample t-test was used to compare groups. To represent non-normally distributed data, the median (quartile) was used, and the Mann-Whitney test was used to compare groups. The x2 or Fisher's exact test was used to compare groups of disordered classification data, and percentage was used for classification count data. The Mann-Whitney test was used to compare groups of ordered taxonomic data.

Pearson correlation was used to analyze the relationship between PA and UT after surgery. 1> r >0 was found to be positively correlated, -1< r <0 was found to be negatively correlated, r=0 was found to be uncorrelated, and P 0.05 was found to be statistically significant.

## Results

Table 1 describes all patient demographic profiles, together with CC condition status at the time of the investigation.

**Table 1:** Overall demographic data for the study cohort (n=233).

Item		Individuals	Percentage（%）
Age (years)			
＜30		17	7.30
30-50		156	66.95
＞50		60	25.75
Marital status			
Unmarried		6	2.58
Married		215	92.27
Divorced		8	3.43
Widowed		4	1.72
Type of medical insurance			
Urban		107	45.92
Rural		126	54.08
Cancer staging			
Ⅰ_b1_		43	18.45
Ⅰ_b2_		91	39.06
Ⅱa_1_		75	32.19
Ⅱa_2_		24	10.30
Pathological type			
Squamous cell carcinoma		198	84.98
Adenocarcinoma		35	15.02
Complication			
Urinary retention		9	3.86
Urinary system infection		13	5.58
Systemic infection		10	4.29
Abdominal incision infection		6	2.59
Length of hospital stay (weeks)	≥1	91	39.06
	<1	142	60.94

Table 2 provides a description of UT scores for the study cohort, involving patients recovering from CC surgical interventions. The mean score value across all items ranged between 3.74 and 4.34 on the UT scale.

**Table 2:** UT scores for patients post-CC surgery (n=233). Data is represented as mean ± SD. UT: Uncertainty tolerance; CC: cervical cancer

Dimension	Point Range	Item Mean Score		Total Score
Powerlessness caused by uncertainty	8～40		4.34±0.52	34.73±4.15
Accidents are negative	9～45		4.24±0.48	38.19±4.30
Pressure caused by uncertainty	6～30		4.06±0.47	24.39±2.84
An uncertain future is unfair	4～20		3.75±0.43	15.02±1.72
Uncertainty Tolerance	27～135		3.74±0.34	101.06±9.18

PA scores were also compiled for such patients (Table 3). The mean scoring for items on this scale across the study cohort ranged between 1.96 and 2.71 (Table 3).

**Table 3:** Positive acceptance scores for the study cohort of post-CC surgery patients (n=233). Data is represented as mean ± SD. CC: Cervical cancer

Dimension	Point Range	Item Mean Score	Total Score
Acceptance	11～55	2.71±0.30	29.91±3.32
Positive	14～70	1.37±0.21	19.30±2.90
Positive acceptance	25～125	1.96±0.20	49.20±4.88

**Figure 1:**
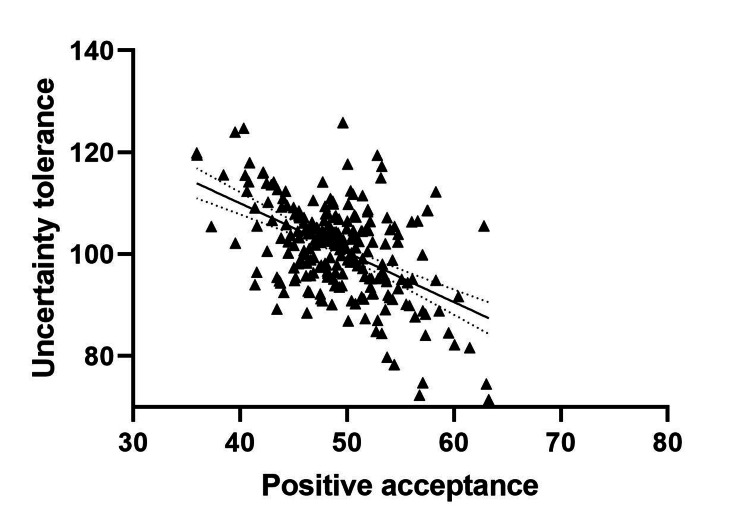
Correlation between UT and positive acceptance in patients with CC (post-surgery). UT: Uncertainty tolerance; CC: cervical cancer

Table 4 represents the findings for possible correlation between UT and PA of patients, post-CC surgery. The UT for patients post-CC surgery was negatively correlated with PA (r=-0.432, P<0.05). Graphical representation of this correlation is reflected in Figure 1.

**Table 4:** Correlation between UT and positive acceptance (r value). Note: * is P＜0.05, * * is P＜0.01 UT: Uncertainty tolerance

Variable	Acceptance	Positive	Positive acceptance
Impotence caused by uncertainty	-0.242^﹡﹡^	-0.360^﹡﹡^	-0.379^﹡﹡^
The pressure of uncertainty	-0.326^﹡﹡^	-0.268^﹡﹡^	-0.381^﹡﹡^
Accidents are negative	-0.354^﹡﹡^	-0.378^﹡﹡^	-0.466^﹡﹡^
An uncertain future is unfair	-0.407^﹡﹡^	-0.305^﹡﹡^	-0.459^﹡﹡^
Uncertainty Tolerance	-0.391^﹡﹡^	-0.420^﹡﹡^	-0.432^﹡﹡^

Table 5 depicts the results of comparative analysis of UT scores in CC patients having differing different lengths of hospital stays. The UT score of postoperative CC patients with the length of stay ≥1 week was significantly higher than that of patients with the length of stay <1 week (P<0.05) (Table 5).

**Table 5:** Comparison of UT scores of CC patients with differing lengths of hospital stay. Note: t is the t-test value; P is the P value. UT: Uncertainty tolerance; CC: cervical cancer

Dimension	Length of stay (weeks )	Point Range	Mean Item Score	Total score
Powerlessness caused by uncertainty	≥1 (n=91 cases)	8～40	4.39±0.54	36.25±4.20
	<1 (n=142 cases)		4.11±0.46	33.62±4.16
t			4.232	4.691
P			0.000	0.000
Accidents are negative	≥1 (n=91 cases)	9～45	4.29±0.50	39.35±4.32
	<1 (n=142 cases)		4.08±0.46	36.29±4.38
t			3.286	5.231
P			0.001	0.003
Pressure caused by uncertainty	≥1 (n=91 cases)	6～30	4.13±0.48	24.85±2.85
	<1 (n=142 cases)		3.91±0.45	22.88±2.85
t			3.547	5.148
P			0.001	0.000
An uncertain future is unfair	≥1 (n=91 cases)	4～20	3.81±0.45	15.81±1.73
	<1 (n=142 cases)		3.61±0.44	14.32±1.67
t			3.355	6.552
P			0.001	0.001
UT	≥1 (n=91 cases)	27～135	3.81±0.36	116.64±8.15
	<1 (n=142 cases)		3.56±0.45	103.04±9.67
t			4.462	11.120
P			0.025	0.000

Table 6 depicts the results of a comparative analysis of PA scores in CC patients (post-surgery) with differing lengths of hospital stay. The score of PA in patients with postoperative CC - whose hospital stay was ≥1 week - was significantly lower than for patients with a hospital stay <1 week (P<0.05) (Table 6).

**Table 6:** Comparison of positive acceptance scores for post-surgery CC patients with differing lengths of hospital stay. Data is represented as mean ± SD. Note: t is the t-test value; P is the P value. CC: Cervical cancer

Dimension	Length of stay (weeks )	Point Range	Item Mean Score	Total Score
Acceptance	≥1 (n=91 cases)	11～55	2.34±0.24	27.95±2.56
	<1 (n=142 cases)		2.75±0.31	30.25±3.36
t			11.332	5.910
P			0.000	0.000
Positive	≥1 (n=91 cases)	14～70	1.29±0.16	17.27±2.26
	<1 (n=142 cases)		1.48±0.22	20.16±2.54
t			7.118	8.840
P			0.000	0.000
Positive Acceptance	≥1 (n=91 cases)	25～125	2.09±0.21	49.55±6.38
	<1 (n=142 cases)		2.35±0.28	51.90±6.45
t			7.593	2.725
P			0.000	0.007

**Figure 2:**
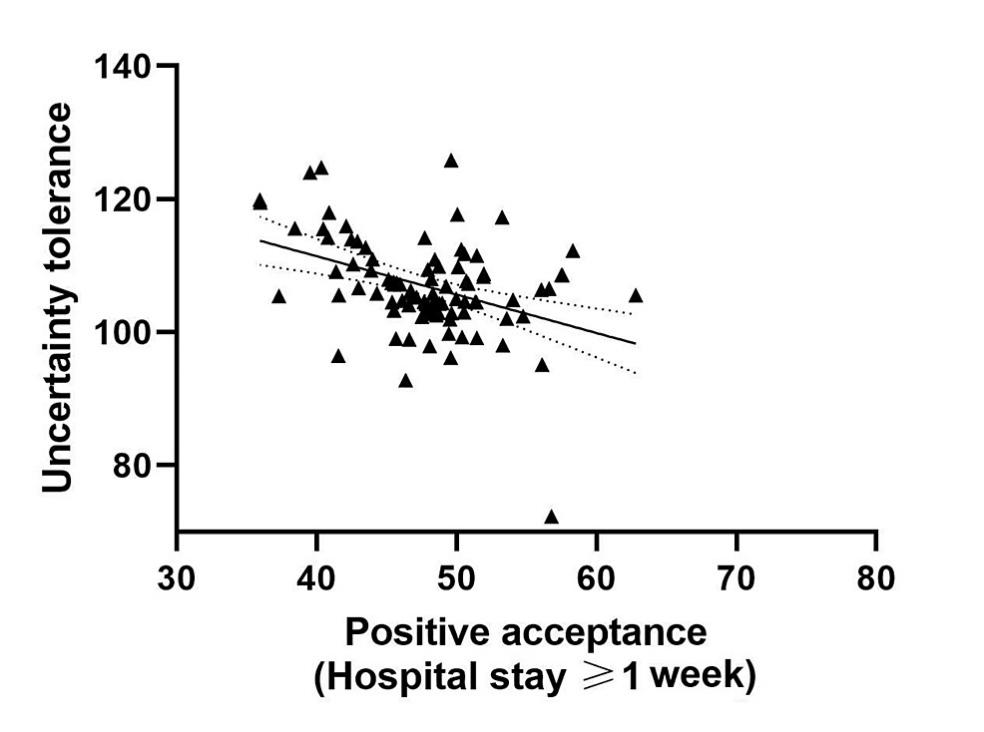
Correlation between UT and positive acceptance in post-operative CC patients with hospital stays ≥ 1 week. UT: Uncertainty tolerance; CC: cervical cancer

**Figure 3:**
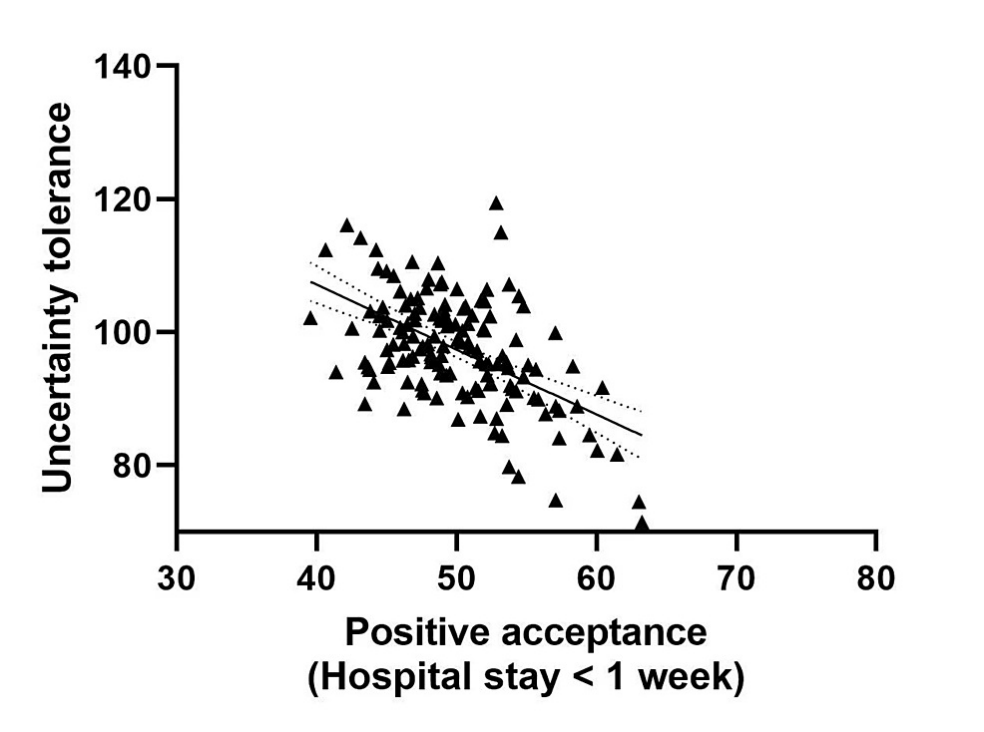
Correlation between UT and positive acceptance in post-operative CC patients with hospital stays < 1 week.

Table 7 describes the results for possible correlation between UT and PA in post-surgery CC patients with differing lengths of hospital stay. The UT of patients with hospital stays < 1 week was negatively correlated with PA (r=-0.358, P<0.05). UT was negatively correlated with PA in patients with hospital stay ≥1 week (r =-0.493, P<0.05). Increased hospitalization time was linked to a raised UT score and a reduced PA score (see Table 7, Figure 2 and Figure 3).

**Table 7:** Correlation between UT and positive acceptance in post-surgery CC patients with differing lengths of hospital stay (r value). Note: ** is P＜0.01. UT: Uncertainty tolerance; CC: cervical cancer

Parameter	Length of stay (weeks)	Positive Acceptance	
		≥1 (n=91 cases)	<1 (n=142 cases)
UT	≥1 (n=91 cases)	-0.493**	-
	<1(n=142 cases)	-	-0.358**

## Discussion

Studies have shown that UT has a significant impact on cancer and non-cancer groups [6]. Different lengths of hospital stay may also affect patient UT. CC cases recovering from surgery-intervened CC exhibit greater levels of acceptance and positivity, as indicated by their greater scale scores. The results showed that the UT score of post-operative CC patients was 4.10±0.48, close to the peak value of 5, indicating that post-operative CC patients had a low UT. In addition, the score of patients with hospitalization duration ≥1 week was significantly higher than that of patients with hospitalization duration <1 week. Consequently, the patient will probably feel unsure if the course of therapy, prognosis, or conclusion of the illness is not foretold. In a comparable manner, the patient's sense of control is inadequate when they experience physical discomfort and lack of knowledge, thus exacerbating UT [9]. Dimensional "inability caused by uncertainty" received the foremost score; this was partially due to a number of uncontrollable circumstances, including delayed recuperation within the initial post-operative phase, worries concerning possible tumor metastases and relapses, and apprehension about possible adverse effects from chemotherapy and radiation, together with prohibitive medical-care costs [10]. Even with innovative therapies, cancer remains one of the most prevalent causes of death globally, and it is challenging to pinpoint the precise prognosis of the illness. The rate of successful treatment for CC patients is greater than that of other aggressive tumors, though, and it might rise exponentially in the future, as medical knowledge grows, vaccinations are used, and novel treatments are developed [11]. Consequently, according to this study's observations, the scale "an uncertain future is unfair" has a low score. The study's findings validated the illness uncertainty theory put out by American nursing scientist Mishel. Alterations in condition UT are also associated with the prevalence and development trend of life-threatening illnesses and are linked to extended hospital stay [12].

Accepting and enterprising attitudes can help adjust the adaptability of individuals’ experiences toward stressful events and may maintain a positive mental state [13]. CC brings great pain to the patient's body and psychology and induces negative emotions such as worry, fear, and low self-esteem [14]. The score of satisfaction and aggressivity of patients post-CC surgery was 2.04±0.26, close to the low peak value 1, indicating that the satisfaction and aggressivity of patients after CC surgery were low, also suggesting that the satisfaction and aggressivity of patients with hospital stay ≥1 week were significantly lower than that of patients with hospital stay <1 week. The low score of "Yes" in the dimension is related to CC, a malignant tumor unique to women. This may partly be due to multiple factors, such as personal privacy, sexual life history, daily habits, behavior of sexual partners, and husband, and wife relationships, among other factors. Consequently, the psychological pressure on CC patients is higher than for patients with other cancers [15]. There are negative effects such as family life chaos, self-career obstruction, and social role loss, combined with the side effects of post-operative radiotherapy and chemotherapy, as well as the extension of hospital stay. Such patients have negative emotions, which will lead to a low degree of self-acceptance. The high score of “enterprising” in the dimension is related to the strong resilience of Chinese women, the desire for business, the sense of family responsibility, the willingness to express emotions to obtain social support, actively seeking treatment information, and cooperating with medical staff.

The statistical results showed that the score of UT of patients post-CC surgery was negatively correlated with the score of PA, suggesting that reduced UT in patients to the event, was linked to a reduced level of PA. The length of hospital stay post-CC surgery was typically one week. Following extended hospital stays, the UT score of patients post-CC surgery was significantly worse than that of patients with hospital stays≥1 week. The UT of patients with hospitalization duration ≥1 week was negatively correlated with PA of patients with hospitalization duration ≥1 week. Patients' UT and PA following CC surgery may be worsened. Prolonged hospital stay also means that patients' recovery ability may be poor, and poor tolerance of uncertainty and PA also lead to increased psychological pressure and slow recovery of patients. The study by Anderson et al. [16] also demonstrated that patients' tolerance of uncertainty varies with length of stay; this agrees with the findings of this investigation. Post-operative patients face the loss of fertility caused by reproductive organ resection, changes in the quality of sexual life, early menopause, the potential threat of residual cancer cells, high medical expenses, and other problems striking women, which may account for this [17]. During the treatment, the patient’s sense of control and self-esteem are relatively low, and the mentality of powerlessness and resignation is more prominent. Studies have shown that individuals with a low tolerance for uncertainty also have higher levels of anxiety and depression [18]. In terms of cognitive-behavioral responses, individuals with a low tolerance for uncertainty hold a pessimistic and skeptical attitude toward the future, over-perceive future uncertain situations, events, or negative news about diseases, and tend to be susceptible to diseases or surrounding events. These patients are also likely to negatively interpret the information [19]. An increase in uncertainty information about the prognosis of CC enables individuals with low UT to be aware of the existence of physical and psychological stress, which may increase their happiness levels. The degree of enterprising is low. This was also confirmed in the work of Turner et al. [20], in which the acceptance factor, the aggressiveness factor, and the total score of positive acceptance initiative were significantly positively correlated with positive coping. There was a significant negative correlation between acceptance factor, enterprising factor, and total score of acceptance /enterprising with depression and anxiety. Aggressiveness and acceptance factors had positive and negative relationships with overall acceptance score, physiological and emotional status, as well as with social and familial status, and functional status. The positive coping factor and acceptance factor all had positive predictive effects on quality of life, according to a linear regression study. The quality of life of CC patients improves with increased PA.

This study does have limitations, namely, in the form of a relatively low cohort size and based solely on a single medical institute. In addition, patient subjectivity, mainly due to psychiatric conditions pre-existing prior to CC development, could have introduced bias in responding to such surveys.

## Conclusions

This study shows that clinical nurses should establish a systematic psychological assessment system, together with personal analysis of CC patients with the specific reason for the UT and PA, enterprising, in order to provide targeted health education, improve the patient's tolerance of uncertainty, be acceptable to promote progressive levels, and reduce the risk of in-hospital time extension, thus helping them to get a higher quality of life.
